# Searching for rich stories in Rich Pictures: Analysing visual stories about living with cancer

**DOI:** 10.1371/journal.pone.0348112

**Published:** 2026-05-12

**Authors:** Emily R. E. Evans, Niels van Poecke, Hanneke W. M. van Laarhoven, Esther Helmich

**Affiliations:** 1 Department of Medical Oncology, Amsterdam University Medical Center, Location University of Amsterdam, Amsterdam, The Netherlands; 2 Cancer Center Amsterdam, Cancer Treatment and Quality of Life, Amsterdam, The Netherlands; 3 Amsta Healthcare Organization, Amsterdam, The Netherlands; Macau University of Science and Technology, MACAO

## Abstract

Stories play an important role in making meaning of illness experiences and (re)establishing self-identity. Yet, telling and investigating stories about living with incurable cancer may be challenging, as people may perceive their illness experiences as hard to communicate. Visual research methods are increasingly employed to tell stories about illness. Rich Pictures (RPs) – hand-drawn, visual stories – stand out due to their ability to visualise and investigate multidimensional illness stories in a single snapshot and, when repeatedly used, their construction over time. RPs therefore present opportunity in investigating the construction of stories about life with an incurable disease. However, RP analysis methods often prioritise the accompanying verbal narratives, and practical guidance for the analysis of RPs themselves remains scarce. This may overlook the valuable contributions RPs can have in understanding illness narratives. As such, we developed a multi-level, systematic analysis process for RPs themselves, i.e., without the analysis of interviews, as stories of life with incurable cancer. Our method comprises a core analytical level – a six-step, systematic coding process, with two ‘extensions’ for comparisons of RPs across different timepoints and groups. This is supported by ‘gallery walks’ – a participatory approach to visual analysis. In doing so, we forward our method and the epistemic richness of analytically engaging with RPs as valuable to research on the ways people living with incurable cancer (re)create their stories, and in research contexts beyond.

## Introduction

Visual representations of illness experiences may be powerful for exploring and narrating stories about life with incurable cancer. Stories are increasingly recognised as fundamental to processes of meaning-making and identity formation when incurably ill [1–3]. Questions about how meaning and identity may be both disrupted and (re)constructed in narrative ways have thus become important lines of inquiry within illness research. Whilst conventional verbal narrative methods (e.g., the semi-structured interview) have crucial value when exploring these questions, illness experiences may also involve phenomena – such as complexity, abstraction, and non-linearity – perceived as difficult to narrate through words alone [4–6]. Consequently, qualitative health researchers increasingly see such approaches as generating sometimes limited insights and analytic avenues into illness, prompting calls for methodological innovation [5,7–9].

Visual methods have been increasingly used – predominantly in partnership with verbal methods – to help express and understand illness narratives [5,10–13]. Among these methods are so-called ‘Rich Pictures’ (RPs) – hand-drawn, free-form, and participant-generated visual stories. RPs are suggested to depict illness stories in a single and holistic snapshot, whilst offering modes of storytelling that can be sensitive towards multidimensionality, abstraction, and non-linearity [4,14–16]. A single RP may therefore assist in narrating illness experiences otherwise difficult to articulate and provide insights into an individual’s narrative meaning-making and identity formation at a specific moment [4]. Alongside this, repeated RPs have potential in capturing the process of narrating illness experiences over time, offering insights into how narrative meaning and identity may be (re)constructed longitudinally, across illness trajectories and care interventions [14,17,18]. As such, RPs may be considered a valuable method for investigating illness narratives in both research and clinical settings [4,14].

In the analysis of RPs, however, researchers have placed different emphases on the drawings themselves and on participants’ subsequent verbal narratives and interpretations. Although weight can be given to both the visual and verbal narratives, precedence is commonly given to the verbal – a tendency observed within visual methods more widely [11,15,19,20]. In this context, RPs may be considered primarily as discussion aids intended to illustrate and facilitate richer verbal narratives, rather than as autonomous narrative material [15,21]. Emphasising verbal data is also often highlighted as a way of addressing the highly interpretive process involved in the analysis of visuals [5,15,16,20–22]. Researchers may thus rely on verbal data as a ‘neutralising’ or ‘gatekeeping’ approach to avoid problems of misunderstanding and mis- or over-interpreting visuals [5,15,16,21,23].

However, RPs themselves, understood as visual illness narratives in their own right, may serve as valuable sources of knowledge that augment understanding and make particular contributions to illness narrative research [13,21,24]. More specifically, within a narrative theoretic perspective, visual storytelling is *also* an important site of meaning‑making and identity construction – inviting analytic attention to the RPs, too. Insights may thus be at risk of being overlooked by relying on the participants’ verbal narratives and interpretations of the RPs and by not exploring how to infer results from engagement with the visual narrative itself [20,24]. Important conceptual approaches to the analysis of visuals – and particularly a constructivist understanding wherein meanings are viewed not as fixed or singular, but as situated, co-constructed, and multiple – may further heighten this concern [11,25]. From this perspective, verbal primacy may not only risk ‘missing’ the visual story itself but incongruently privileges the ‘authority’ of one voice over the productive potential of multiple interpretations [11,26]. Together, these concerns warrant reorientation in the analysis of RPs to bring the visuals themselves into analytical focus.

Yet, there is a pronounced ‘dearth’ of academic attention and practical guidance on the step-by-step processes for the analysis of participant-generated visuals, with even general descriptions largely missing [11,13,21,22,27]. We, therefore, respond to the call for practical support for, and greater transparency in, researchers’ analytical engagement with RPs as a means to advance the potential of this visual narrative method in illness research [5,11,12,19]. To do this, we first briefly introduce RPs before presenting and then reflecting upon a constructivist, multi-level and systematic analysis process for RPs themselves, as visual narratives – examined at one moment, longitudinally, and across groups and care interventions. This approach was developed and implemented in the project ‘In Search of Stories’ (ISOS) [28,29].

## What are Rich Pictures?

**Fig 1:**
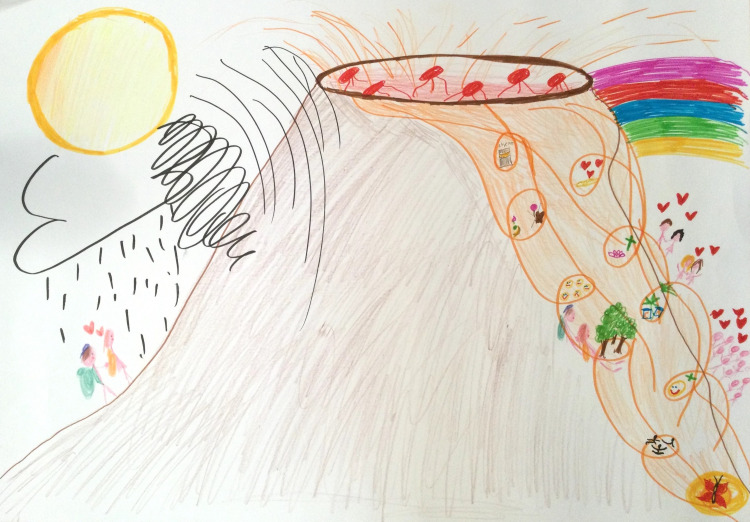
Example Rich Picture about life with incurable cancer from project ‘In Search of Stories’.

RPs were first developed as part of the Soft Systems Methodology to help unravel and plan action on problems within complex systems [22,24,30]. They have since been applied in various domains and contexts, developing as a valuable, independent method – including within illness narrative research [30,31]. RPs are hand-drawn, visual narratives created by research participants, either individually or in groups, to depict complex issues, situations, or experiences (Fig 1) [30]. RPs aim to invite rich stories by encouraging participants to draw all relevant elements while using minimal text [21,30,31]. The resulting visual narratives may explore multiple contributing dimensions from the perspective of the participant(s), such as what happened, material and human actors involved, emotions, behaviours, thoughts, relationships, and processes [4,14,15].

RPs do this without requiring specific artistic skill [30]. Often characterised by their artistic simplicity, RPs have come to be likened to childhood doodles or cartoons [30–32]. However, RPs can tell stories in ways that vary widely, ranging from artistic drawings to stick-figure diagrams [15,31]. Importantly, there are no right or wrong ways to create RPs: they are non-directive and do not follow a prescribed narrative format, providing considerable flexibility to participants when telling stories [21,31]. Every application of RPs is thus considered to vary contingent upon its use, the user, and context [31].

To create RPs, participants are typically provided with paper, coloured pencils or pens, a prompt, and a timeframe (for example, 30-minutes for an individual, and 90-minutes for groups). By requiring so few resources and no specific training or skill, RPs are a potentially low-resource and accessible visual narrative method [31].

## Rich Pictures in search of rich stories: Development of a multi-level analysis approach

**Fig 2:**
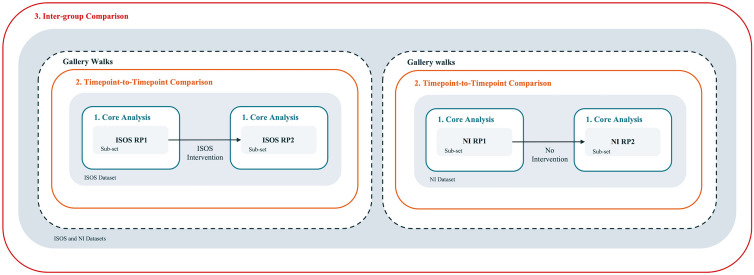
Overview of the multi-level analysis process for RPs themselves.

We developed a multi-level, systematic analysis process for RPs as autonomous narrative material distinct from verbal narratives and informed by a constructivist epistemological perspective. In doing so, we looked to explore the contributions RPs might make to understanding the (re)construction of stories about life with an incurable illness at one moment in time and across time, groups and interventions. Our resulting approach has several analytical levels, building from one core analysis level and inferring results from engagement with RPs themselves (Fig 2). The findings from using this analysis process are published elsewhere [33]. The remainder of this article continues with a specifically methodological focus, guiding researchers through our analysis levels in a distinctly step-by-step reporting style to address field-level demands. It then reflects upon the opportunities and limitations of the multi-level approach itself, and its presentation. By doing so, we aim to propose potentially useful guidelines for researchers who wish to conduct rigorous analyses of RPs themselves in new contexts – whether individually, in groups, over time, or across groups.

### In search of stories: Study context

The analysis process was developed within the context of the project ISOS, a multi-modal intervention aimed at supporting and investigating the (re)construction of stories about life with incurable cancer using arts-based methods [28,29,34,35]. The intervention involved participants co-creating artworks with artists, reading selected literature, participating in life-review interviews, and drawing RPs at two different timepoints: ISOS Rich Picture 1 (ISOS RP1) at the beginning of the intervention (n = 25), and ISOS Rich Picture 2 (ISOS RP2) at its end (n = 12). A preparatory study for ISOS (the ‘No Intervention’ [NI] study) piloted the repeated use of RPs with a separate group of individuals living with incurable cancer. These participants drew RPs at two different timepoints and without further intervention: NI Rich Picture 1 (NI RP1) before a two-month interval (n = 13), and NI Rich Picture 2 (NI RP2) after (n = 13) [4,14]. Together, these studies provided the RP corpus with which we could develop our analysis process. Recruitment processes and inclusion for these studies are described elsewhere and took place between 1st March 2021 and 1st August 2022 (ISOS) and 1st February 2018 and 1st May 2018 (NI) [4,14,35].

### Overview of the multi-level analysis process

Our analysis process consisted of one core analytical level, upon which two possible ‘extensions’ were built, and was supported by a participatory analysis approach (see Fig 2). The core analysis involved a systematic coding process that progressed from granular-level coding to theoretical insights, engaging with RPs in six steps. Building upon insights from this core method, the extension levels addressed our specific areas of interest. First, we developed a timepoint-to-timepoint comparison (T-T comparison): a method for comparing RPs drawn over time. Using this method, we compared ISOS RP1s with ISOS RP2s; or NI RP1s with NI RP2s. Second, we developed an inter-group comparison: a method for comparing two different sets of RPs drawn over time. This facilitated comparison of the intervention group’s RPs (ISOS RP1s and ISOS RP2s) with the non-intervention group’s RPs (NI RP1s and NI RP2s). A participatory analysis method was introduced through ‘gallery walks’, informing and enriching the core analysis and ‘T-T’ comparison. This overall process was underpinned by a constructivist epistemological understanding, situating meaning- and knowledge-generation in context, and as co-constructed by the participant, audience (gallery walk attendees), image, and researcher [11].

### Analysis team, software, and coding terms

Analysis was led by a researcher specialising in participatory arts-based research in oncology (EE), with a background in the arts and sociology. It was supported by a second coder (MP), and a supervisory research group (NvP, HvL and EH), whose backgrounds span qualitative and RP research, the sociology of health and illness, cultural sociology, elderly care medicine, medical education, medical oncology, aesthetics and theology. Gallery walk participants also contributed to the analytic process (see description of gallery walks for detail). Together, the second coder, supervisory group, and gallery walk participants supported the analysis by challenging and extending interpretations through different perspectives, with the aim of enhancing interpretive richness and reflexivity, rather than (quantitatively) standardising findings (e.g., through intercoder reliability measures or member checking) [11,26,36,37]. The supervisory group also supported the development of the analysis method.

We utilised the qualitative analysis program, MAXQDA (version 22.1.1). Though perhaps more typically used for text-based analyses, MAXQDA enables users to code visual data by drawing ‘frames’ around specific pictorial segments – similar to taking selective screenshots – to create coded data segments. Within this context, coded data segments were thus pictorial segments of RPs. Mirroring the structure in MAXQDA, our code hierarchy throughout this article reads from left (highest) to right (smallest). ‘Parent’ and ‘subcodes’ denote code hierarchy, while, for example, ‘preliminary category’, ‘granular code’, and ‘descriptive code’ denote code type (Table 1).

**Table 1:** Code glossary. Table 1 presents a glossary of codes used throughout our core and ‘T-T’ coding processes.

Core analysis				
*Code type*	(Preliminary)/major category	Intra/inter-category interpretative	Descriptive	Granular
*Example*	Nature	Being in nature as meaningful activity	Smiling couple sitting by trees	Tree(s)
*Coded data segments*	All pictorial segments relevant to category	All pictorial segments relevant to interpretative code	Pictorial segment showing described scene	Pictorial segment limited to the corresponding individual icon or piece of text only
*Generated in step*	(2)/5	4/5	3	1
**T-T analysis**				
*Code type*	Inter-participant	Participant-specific		
	Major comparative category	(Preliminary comparative category)	Code-assisted comparative	Visual comparative
*Example*	Reinforcing	(Reinforcing)	Small supportive role of relations → large supportive role of relations	People smaller → people larger
*Coded data segments*	Pictorial segments across all RPs relating to strategy of reinforcement	All pictorial segments of participant-specific RPs relating to category	Pictorial segments of participant-specific RP1 and RP2 being compared	Pictorial segments of participant-specific RP1 and RP2 being compared
*Generated in step*	4	3	2	1

Following MAXQDA’s coding hierarchy, highest level (parent) codes are positioned on the left, while subcodes get smaller as they read to the right. However, consistent with our inductive approach, the steps described in the remainder of our manuscript generate codes progressively from right to left (with exclusion of preliminary categories in the core analysis). Code types marked with parentheses highlight their particularly preliminary nature.

### Ethics

The study was exempted from ethical approval by the Medical Ethics Review Committee of the Academic Medical Center and Amsterdam University Medical Center, since the Medical Research Involving Human Subjects Act was not applicable (reference number: W20_436 # 20.483; W17_476 # 17.549; 2024.0319). Written informed consent was obtained at the start of participation from those drawing RPs and included in gallery walk 2. Oral informed consent was obtained from academic peers, prior to and at the start of gallery walk 1.

## Core analysis: A systematic coding approach to RPs

**Fig 3:**
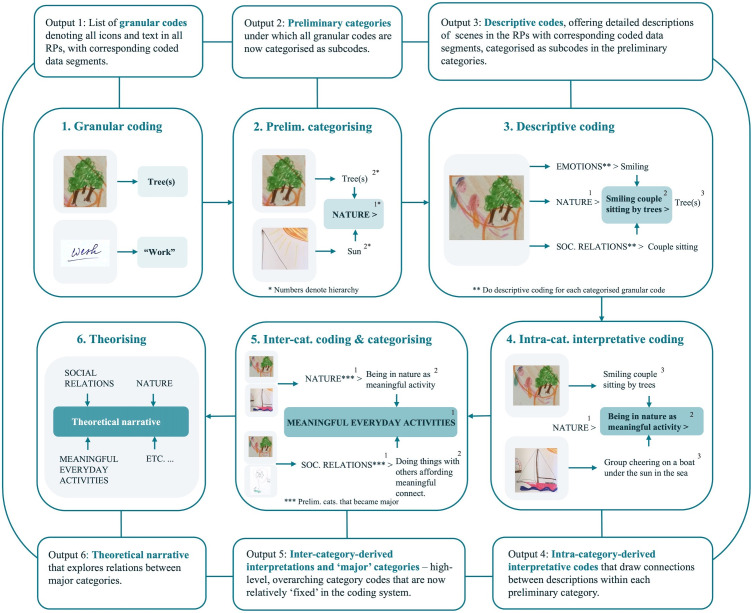
Core analysis method as a systematic coding process for RPs.

In this first section, we describe the development of the core analysis method as a systematic coding process for RPs. This process begins with granular, icon-by-icon coding and moves towards making theoretical observations about, and through engagement with, visual narratives about living with incurable cancer drawn at one moment in time (see Fig 3). It is the foundation for further analytical levels.

Our coding approach is inspired by inductive approaches used in verbal data analysis, in which analysis tends to all aspects of the data towards grasping both the implicit and explicit dimensions of participants’ narratives [13]. We analysed each subset – ISOS RP1s, ISOS RP2s, NI RP1s and NI RP2s – independently through this method, generating four different code sets in the same overall coding system.

### Core analysis: The six coding steps

#### Step 1: Granular coding.

All individual visual icons, or pieces of text, appearing in all RPs were first coded with ‘in vivo’ codes. We understood these codes to be granular codes generated ‘directly’ from all discursive icons and text, sticking as close to the data as possible and resisting further analytical interpretation. For example, an icon of a tree or “work” as text in a RP, became ‘tree’ and “work” as separate granular codes (see Fig 3, step 1; and Table 1). Single quotation marks indicated an icon or visual motif, whereas double quotation marks indicated when a code referred to text. Any unidentifiable or unreadable icons or text were provisionally coded as ‘ambiguous icon or text’ for later review, usually with other members of the research group or gallery walk participants. Coded data segments were limited to the corresponding individual icon or piece of text only, and not yet inclusive of other icons in the same RP. This step generated an extensive list of granular codes denoting all icons and text included across all RPs, attached to corresponding visual/textual data segments.

This kind of coding may appear mundane. However, the size of the unit of data coded in the analysis is crucial, and beginning with the ‘mundane’ in visual analysis – as in textual analysis – can be particularly useful [27,38]. The aim of coding in this way was to heighten attention to all discrete visual/textual units of data in every RP. We found it generated extremely close engagement with the visual narratives – along with an extensive list of all the icons and text within the RPs. Together, these enabled exploration of how participants constructed their visual narratives at a granular, iconographic level.

#### Step 2: Preliminary categorising.

Next, we grouped the granular codes into preliminary categories. Preliminary categories were not predetermined and were generated from engagement with the granular codes. I.e., the granular codes ‘tree’ and ‘sun’ were grouped into the preliminary category of ‘Nature’ (see Fig 3, step 2). No further interpretations were made. This step generated a list of preliminary categories, under which all granular codes were now categorised.

In the coding system, granular codes were organised as subcodes, with preliminary categories as parent codes (see Table 1). This made a two-level coding hierarchy: ‘Preliminary category [parent code]> granular code [subcode]’, e.g., ‘Nature [parent code]> tree [subcode]’.

The initial intention for this step was practical: to make the coding system more manageable. Yet, integrating this step allowed us to form a preliminary overview of what was granularly appearing in the RPs and create structure for further analysis. Though preliminary categories were not necessarily fixed, some remained analytically significant – e.g., ‘Nature’ and ‘Social Relations’ – becoming ‘major categories’ as the analysis developed (see step 5).

#### Step 3: Descriptive coding.

In this step, small ‘scenes’ within the RPs were coded with categorised descriptive codes. Here, attention shifted from the individual icon to several ‘related’ icons within a RP, but not yet to the entire RP (see Fig 3, step 3, for example ‘scene’). This was analogous to shifting from ‘word-by-word’ analytical attention to ‘sentence-by-sentence’. Outputs were descriptive codes offering detailed descriptions of scenes in the RPs, categorised as subcodes in preliminary categories.

To do this step, we began with a categorised granular code, (e.g., ‘Nature> tree’), and observed its coded data segment (e.g., tree icon) within the context of the RP. We considered how this icon related to other icons or text in the same RP, and their corresponding categorised granular codes, to determine a so-called ‘scene’. For example: the immediate ‘scene’ surrounding the tree included icons of two people (coded as ‘Social Relations> couple sitting’) with smiling facial expressions (coded as ‘Emotions> smiling’) (see Fig 3, step 3). We then developed a descriptive code for this scene: ‘smiling couple sitting by trees’. The coded data segment for a descriptive code was the pictorial segment of the RP showing the described scene.

We organised the coding system in a three-level hierarchy: ‘Preliminary category [parent code]> descriptive code [1^st^ level subcode]> granular code [2^nd^ level subcode]’ (see Table 1). For example, ‘Nature> smiling couple sitting by trees> tree’. This structure maintained the preliminary categorisation whilst adding context through the descriptive code.

We created categorised descriptive codes for every categorised granular code, even if repeatedly describing the same scene. This approach ensured rigorous descriptive coding of scenes across multiple relevant categories. For example, we would repeat this step for the categorised granular code ‘Social Relations> couple sitting’, focusing on the icons of the two people in the same scene as the tree, described above. This would create another categorised descriptive code: ‘Social Relations> smiling couple sitting by trees> couple sitting’. Descriptions created when approaching different icons in the same scene did not have to be the same, as one might explore different nuances or emphasis. This promoted multi-perspective descriptions by encouraging us to view scenes within the visual narratives from different angles, such as focusing on nature in one instance, and social relations in another.

#### Step 4: Intra-category interpretative coding.

In this interpretative step, we explored relationships amongst categorised descriptive codes, and their coded data segments, within the same preliminary category. We then developed intra-category-derived interpretative codes that grouped multiple categorised descriptive codes. For example, within the ‘Nature’ preliminary category, ‘smiling couple sitting by trees’ and ‘group cheering on a boat under the sun in the sea’ may come to be grouped under the intra-category-derived interpretative code, ‘being in nature together as meaningful activity’, when the coded data segments were looked at together and within the RPs more generally. Outputs were intra-category-derived interpretative codes that drew connections between descriptions within each preliminary category.

This step expanded our coding hierarchy to four levels: ‘Preliminary category [parent code]> intra-category-derived interpretative code [1^st^ level]> descriptive code [2^nd^ level subcode]> granular code [3^rd^ level subcode]’ (see Table 1). E.g., ‘Nature> being in nature together as meaningful activity> smiling couple sitting by trees> tree’.

As mentioned in step 3, we often descriptively coded the same RP scenes within multiple preliminary categories. This approach helped to now explore multi-dimensional interpretations of the same scene. For example, consider the descriptive codes: ‘Social relations> group cheering on a boat under the sun in the sea’, and ‘Social relations> smiling couple sitting by trees’. These codes describe the same two scenes as the earlier examples in this step, categorised within the preliminary category ‘Nature’ – and yet, here they are categorised in ‘Social Relations’. These were then instead grouped under the intra-category-derived interpretative code, ‘Social relations> doing things with others affording meaningful connection’, when explored alongside other codes and coded data segments within ‘Social Relations’.

#### Step 5: Inter-category interpretative coding and categorising.

Next, we created new or revised categories and inter-category-derived interpretative codes by iteratively exploring connections across all interpretative and descriptive codes in all preliminary categories*.* This process led to the development of different, more abstract and nuanced categories than those generated in step 2, and the identification of so-called ‘major categories’. For example, ‘Social relations> disruption to family’ and ‘Nature> as a metaphor for disruption’ are two interpretations within different preliminary categories that also came under a new, more abstract category of ‘Disruption’. Or: ‘Nature> being in nature together as meaningful activity’ and ‘Social relations> doing things with others affording meaningful connection’ connected under ‘Meaningful everyday activities’ (see Fig 3, step 5).

These new categories became specific lines of enquiry, explored and developed in relation to all the RPs. We also found it insightful here to go back towards the icons and re-examine how these categories were drawn at an iconographic level. Thus, exploration of categories in this step was iterative, working across multiple levels and moving back and forth through the different steps. Importantly, this mobilised the analysis to generate holistic insights tending to both what was being told, and how at a granular level it was being told. Outputs were inter-category-derived interpretations and so-called ‘major’ categories – high-level, overarching category codes that are now relatively ‘fixed’ in the coding system, explored across all other code levels.

#### Step 6: Theorising.

The final step explored the relationships between the major categories to form a theoretical narrative. This entailed looking at the major categories and exploring whether and how they related to one another in the RPs (see Fig 3, step 6). This was done by, for example, selecting major category codes to ‘appear’ in the RPs. This is possible in MAXQDA by first differently colour-coding codes. We could then observe the differently coloured boxes overlaying the RP demarcating particular codes’ coded data segments. This is one example helpful for seeing how, compositionally, major categories (co-)occur across RPs. Throughout our analysis it became clear that major categories were highly entangled with one another – here we could analytically pursue this entanglement. If working with several RP sub-sets or sets, we suggest that this step happens after the collation of code-sets, as described in the next level of analysis.

## Comparative analysis approaches to Rich Pictures

This next section details our two comparative analysis approaches to RPs: (1) the ‘T-T’ comparison exploring narrative changes over time; and (2) the inter-group comparison exploring the impact of interventions on illness narratives. Findings from these analyses, including worked examples can be found elsewhere [33].

### ‘Timepoint-to-Timepoint’ comparison: Coding steps

**Fig 4:**
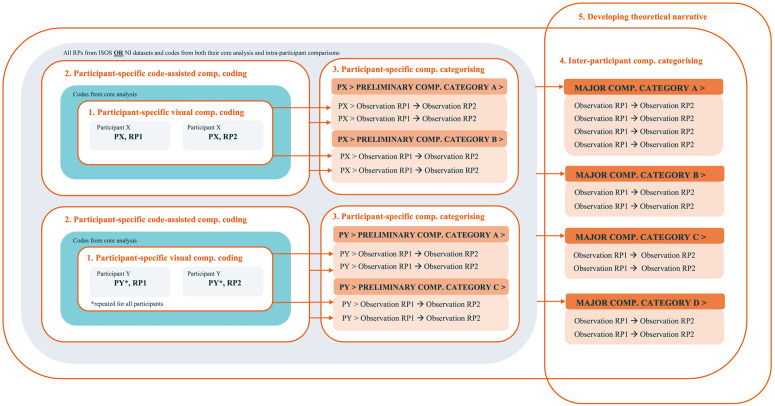
Timepoint-to-timepoint comparison method for the analysis of repeated RPs drawn over time.

**Fig 5:**
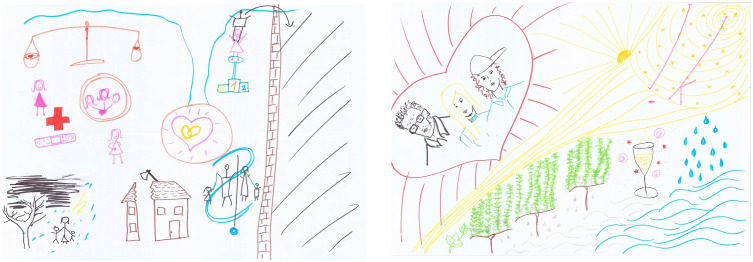
Example RPs drawn over time and across the ISOS intervention (RP1 on the left and RP2 on the right). These RPs, with amendments indicating strategies and as raw files, are published before under the terms of the Creative Commons Attribution License [33].

In this first comparison section, we describe the development of the ‘T-T’ analysis method as a comparative coding process for RPs drawn over time (Fig 4). This process begins with making ‘simple’ visual comparison of individuals’ RP pairs, before drawing upon the core analysis to assist in comparisons, and then moving towards generating higher-level observations about changes in visual narratives about living with incurable cancer over time (Fig 4). Together with the core analysis, this formed the foundation for the inter-group comparison. An example of two RPs drawn over time, and across the ISOS intervention, can be found in Fig 5.

We analysed each set – ISOS RP1s and ISOS RP2s; NI RP1s and NI RP2s – independently through this method, generating two different comparative code sets in the same overall coding system.

### Preparatory steps

Prior to any comparative engagement, we recognised the need for a rigorous understanding of the components being compared. Therefore, the six-step method was first carried out separately for each individual RP-subset. To enable analytical comparability, codes appearing in one set then needed to be looked for in the other sets. We therefore aligned the core analysis code-sets with one another, generating a combined ‘master’ schedule. This was an important step for making robust, and core analysis-assisted, comparisons.

#### Step 1: Participant-specific visual comparative coding.

In this step, we looked at pairs of RPs drawn by the same participant to generate participant-specific comparative codes (see Fig 4, step 1, centre left). Our approach involved a straightforward visual comparison of the RPs – ‘simply’ looking at them together to notice similarities or differences. We coded all visual comparisons we could see with comparative codes. We did not yet explicitly use the codes generated in the core analysis to directly inform comparisons.

This process was executed by simultaneously viewing and coding two RPs by the same participant side-by-side in MAXQDA – e.g., Participant X (PX) RP1 and PX RP2 – with all core analysis codes ‘hidden’. Coding focused on generating comparative codes that remained at the level of describing visual comparisons, without further interpretation. These were written: ‘Observation RP1→ Observation RP2’. For example, ‘people smaller → people larger’. Comparative codes were grouped as subcodes under a parent code referencing a specific participant. The structure of our coding was therefore: ‘Participant X [parent code]> Observation RP1→ Observation RP2 [subcode]’ (see Fig 4). Coded data segments were the pictorial segments of RP1 and RP2 being compared.

The aim of returning to ‘just’ visually looking at the RPs and describing our visual comparisons was to embed our comparative analysis in engagement with the visual narratives themselves.

#### Step 2: Participant-specific code-assisted comparative coding.

In this step, we continued with the RPs drawn by the same participant but looked to further our comparative coding by explicitly mobilising the core analysis coding (see Fig 4, step 2).

We found different approaches enabled us to use the core analysis in making comparisons. In doing so, we compared: (1) *an exported code overview of each RP* – this provided a broad perspective on the distribution of codes (major categories, interpretations, descriptions, granular) from the core analysis across the two RPs, useful for identifying overall shifts and similarities, and serving as a good orienting step for further analyses; (2) *how codes visually ‘appeared’ in the two entire RPs* – this generated insights into the arrangements/compositions, sizes, and so forth of codes from the core analysis in the context of the RP; (3) *what and how specific codes appeared across two RPs* – this allowed for focused examination of how particular codes (dis)appeared across RPs; and (4) *specific codes’ coded data segments* – allowing for exploration of the specific details and nuances of pictorial segments across RPs, useful for detecting subtle differences in how certain categories and interpretations were depicted. By employing these multiple, different code-assisted comparative approaches, we were able to achieve a multi-faceted comparative understanding of the data, developing and pursuing the diverse enquiries that come up in the comparison process, and gaining both broad and very detailed perspectives on the similarities and differences between RPs.

This step generated new comparative codes and embedded comparative codes in the core analysis insights. It thus often took comparisons from a descriptive to interpretative level. For example, by looking at the core analysis codes, we noticed that whilst the major category ‘Nature’ remained in the RP [‘PX> RP1 → RP2 nature remains’], there was a shift in its use. This generated the new code, ‘PX> nature as metaphor for disrupture → nature as metaphor for flow’. A second example here would be: in step 1, we observed that people were drawn much more largely in RP2 than RP1. By looking at the codes already attributed to the RP, and the RPs themselves, we could adjust the code to: ‘PX> small supportive role of relations → large supportive role of relations’. Yet, in also comparing the coded segments in detail, we noted a very nuanced shift in what this supportive role looked like.

#### Step 3: Participant-specific comparative categorising.

In this next step, we focused on each participant’s comparative codes individually (i.e., not in relation to other participants’ codes), aiming to create preliminary comparative categories (see Fig 4, step 3). In this step, we observed shifts not only in *what* people were telling (i.e., changes in major categories from the core analysis) but also began to identify strategies in *how* these changes were occurring. For example, ‘PX> small supportive role of relations → large supportive role of relations’ and ‘PX> small use of nature → nature as dominant’ started to develop a preliminary comparative category of ‘Reinforcing’ in the use of both relations and nature.

Our code hierarchy here became: ‘Participant X [parent code]> Preliminary comparative category [1^st^ level subcode]> Comparative code [2^nd^ level subcode]’. For example, ‘PX> Reinforcing> small supportive role of relations → large supportive role of relations’.

#### Step 4: Inter-participant comparative categorising.

We then shifted focus from individual participants to developing major comparative categories across all participants. This was done by tending to all participant-specific comparative categories and codes together and applying approaches described in step 2 to compare the whole RP1 subset to the whole RP2 subset (see Fig 4, step 4).

First, we moved all preliminary comparative categories and comparative codes out from under the participant-specific parent codes and started to create an ‘overall’ comparative coding system. For example, ‘Reinforcing’ developed in relation to PX now combined with similar preliminary comparative categories or comparative codes from other participants’ RPs, allowing for further development (see the expansion of ‘category A’ in Fig 4, step 4). New comparative categories also emerged here as comparative descriptive codes developed from one participant’s RPs could now be considered next to the comparative codes in another participant’s RPs (see the arrival of ‘grouping D’ in Fig 4, step 4). As comparative categories developed, we would revisit all RP pairs to specifically explore and code them in the dataset. Using comparative approaches described in step 2 and expanding these to whole subsets (comparing the whole ISOS or NI groups’ RP1s with RP2s), helped us to develop emerging enquiries at an inter-participant level. This was carried out until major comparative categories were reached.

Importantly, through this process, we identified significant strategies used for changing narratives across all major categories developed in the core analysis. This was a distinct finding from analytically engaging with the RPs themselves through this delineated method. As this finding became clear, we thus found it useful to organise our coding system as follows: ‘Major Category (e.g., Social Relations) [parent code]> Major Comparative Category (Strategy of reconstruction - e.g., Reinforcing) [1^st^ level subcode]> Comparative code (e.g., small supportive role of relations → large supportive role of relations) [2^nd^ level subcode]. This made it easier to mobilise the core coding for the comparative analysis and effectively captured the ‘whats’, ‘hows’, and descriptions of participants’ visual narratives, useful for further steps.

#### Step 5: Theorising.

Here we looked at the comparative ‘whats’ (major categories) and ‘hows’ (the strategies) (e.g., ‘Social Relations> Reinforcing’, ‘Nature> Reinforcing’) in relation to one another, across all the RPs. This enabled the development of higher-level overviews on the narrative construction of stories about living with incurable cancer over time. As with the core analysis, if continuing to the next extension level, it is helpful to carry this step out after creating the combined comparative schedule.

### Inter-group comparison

In this second comparison section, we describe the inter-group analysis as a comparative process for repeated RPs across different groups. At this level, comparisons between sets were entirely centred upon analytical insights generated in the preceding core and T-T levels, and our approach was no longer explicitly about generating new codes. The intention was to see if we could notice any similarities or differences in the (way) codes generated in these prior levels (were) appearing cross the two sets. To do this, we used the strategies outlined in step two of the T-T analysis, now at a dataset-to-dataset level. As described in the preliminary steps of the T-T comparison, the inter-group comparison began with combining of comparative code-sets into one schedule.

#### Step 1: Inter-group core analysis comparison.

We first compared the core analysis codes across RP1s (i.e., ISOS RP1s with NI RP1s) and RP2s (i.e., ISOS RP2s with NI RP2s) of both sets. This was helpful for pursuing differences in *what* participants in the ISOS group and NI group were telling at each timepoint and understanding how comparable the start and end point RPs were. In doing so, it assisted us in reaching analytical conclusions on how the groups varied in changes in what they were telling about their experiences over time.

#### Step 2: Inter-group T-T analysis comparison.

We then compared the T-T comparisons across ISOS RPs and NI RPs. For example, taking ‘Social Relations> Reinforcing’, or just the strategy of ‘Reinforcing’ across all major categories, we then looked at how (often) and with whom this code was occurring across the two datasets. In doing so, we could start to observe interesting differences in strategy use between the groups. It then became insightful to look at how the co-occurrence of categories and strategies varied across the two groups. We could then create theoretical linkages between how participants were (re)constructing their stories, and the changes in what they were telling. Then, significantly, differences in this between the intervention and non-intervention group.

## Gallery walks: A participatory analysis approach to RPs

Our analysis method was supported by so-called ‘gallery walks’ – a participatory method often used in the analysis of Rich Pictures [4,14,15,31]. Gallery walks are interactive group sessions where participants view and discuss multiple RPs, displayed on walls as if in a gallery [4,14,15]. They can be used as a response to visual data’s potential to have multiple meanings (polysemy), and thus the potential for multiple voices, perspectives, or viewpoints in their interpretation (polyvocality). Gallery walks encourage rich, multi-layered, and intersubjective interpretations of the RPs, as a strategy for capitalising on viewers diverse subjectivities to deepen understanding [4,11,30]. In doing so, these explicitly collaborative interpretations may yield insights that can prompt new lines of analytical enquiry [15]. Moreover, by exploring various RP interpretations, researchers may enhance accountability for their conclusions, the processes that led to them, and remaining gaps or limitations [11]. In these ways, gallery walks may enrich interpretations, inform coding and help to incorporate principles of reflexivity and co-creation in RP research [15].

### Gallery walk participants

Gallery walks can be held with different stakeholders, with the intention that participants bring diverse perspectives contributing to a multi-perspective interpretation of the pictures [4]. In our project, we conducted two gallery walks with two different groups. The first gallery walk was with an interdisciplinary group of researchers (n = 6), involving those in palliative care, the medical humanities, and/or experienced with using RPs. The second gallery walk involved people from the same illness group, i.e., people with incurable cancer, and formal and informal caregivers (n = 6). Generally, stakeholders in gallery walks might include practitioners, researchers, research participants, and family members [15].

Previous research has emphasised the importance of including gallery walk participants who are members of the same (illness) group as the RP creators [4]. This was similarly experienced in our research. These participants – as well as health professionals – promptly recognised elements in the RPs less apparent to researchers, such as embodied, physical sensations, and medical conditions [4]:

GW1 P4: In this I see the side effects of chemotherapy. You see it in the fingers.

GW2 P1: Yes, it’s neuropathy.

Including family members alongside the person with incurable cancer also indicated the usefulness of gallery walks and RPs for exploring the relationality of illness stories [7].

### Gallery walk approaches

Gallery walks are participant-driven and semi-structured sessions. A loose framework is provided by the researcher. This framework may include general steps aiming to assist the group in generating and exploring interpretations together. In our project, these steps were: (1) *an individual and initial viewing of the RPs –* participants were invited to explore the gallery on their own and to form initial impressions of the RPs; *(2) sharing initial observations and impressions on the RPs with the group –* participants were invited to reflect on the initial viewing of the RPs with the group, bringing forward any response they would like to, and continuously interacting with and referring to the RPs whilst doing so; and (3) *exploring and developing interpretations through interactive gallery re-assembly –* participants played the ‘gallery curator’ role by taking down, moving around and clustering RPs in order to develop and discuss patterns, structures, contrasts and commonalities across them. Participants could repeat steps as they liked.

We additionally explored the use of gallery walks in comparing two different subsets of RPs (ISOS RP1 and ISOS RP2). To our knowledge, this is the first attempt to do so. We trialled two different approaches – with both yielding valuable insights.

Approach 1: Combining RP1s and RP2s into a single group (without distinction) for steps 1–3, later inviting participants to exploratively categorise the RPs into ‘before’ and ‘after’ groups. This strategy proved helpful for looking at whether and how participants identified themes across both sets of RPs and for generating exploratory comparative criteria for further data analysis. This was demonstrated by gallery walk 1 participants as they, for example, discussed the removal of depictions of death from the RP1s to RP2s as a provisional comparative criterion [33]:

GW1 P1: I thought that maybe this was the funeral which is why I thought it was pre- the trajectory.

GW1 P3: That is a very interesting question though: when you’re further in the trajectory, do you focus so much on the end? Or is that the first thing that you worry about earlier in the trajectory and then it becomes less of an issue?

Approach 2: Presenting RP1s only for steps 1–3, subsequently ‘revealing’ RP2s for a comparison activity. This approach facilitated more explicit discussions about the differences and similarities between the two data subsets. For example, as gallery walk 2 participants made direct comparisons between two RPs by the same participant [33]:

GW2 P4: [H]er family, friends have come to stand closer around her.

GW2 P6: Yeah, she stands more in the centre [...] They were already close. But there she was less forward, and now she is really in-between them.

Whilst these steps add to the use of gallery walks in making comparisons of RPs from timepoint-to-timepoint, we did not, however, explore making inter-group comparisons in the gallery walk – a task considered unfeasible given the number of rich pictures (n = 63), and level of analytical abstraction. Given that the inter-group analysis focused on mobilising the core and T-T analyses – contributed to by participants in gallery walks – we did not see the lack of an explicitly participatory dimension as compromising. This points to the value of a multi-level approach, while also highlighting the limits of participatory analysis and, in turn, the crucial role of researchers in conducting complex analyses to generate insights into visual narratives [11].

### Gallery walk data collection

We found that gallery walks require comprehensive documentation of both dialogue and visuals. The inclusion of visual data collection is helpful for relating the discussion to the RPs and for capturing how the RPs are clustered and reassembled. In our project, data collection involved multiple methods, including audio recordings, photographs, film, and written notes that captured both textual and pictorial information. These written notes included key themes and remarks responding to the participants’ interpretations, alongside short-hand icons that represented key visual elements explicitly discussed or perceived as significant to the researcher during the discussions. Immediately following the gallery walks, the members of the research team involved (EE & EH) engaged in discussion to reflect on and summarise the session.

### Integration of gallery walks into further analysis

Gallery walks are an analytical approach in and of themselves. However, a step was still needed to be made to systematically draw on their insights to enrich and support the core method and T-T comparison. Generally, the point at which gallery walks are integrated within this kind of analysis can vary. They can be used in the early coding stages, and as theoretical interpretations are developed [15]. We primarily used them from step 3 in the six-step method to assist in the more interpretative steps. Yet, they were also useful to retrospectively (re)identify icons. In the T-T comparisons, gallery walks could inform analysis at several of the steps, as participants made useful visual comparisons (i.e., as with step 1 in the T-T comparison) and useful comparative interpretations for us to pursue in the data. Thus, we see they can be used at various points and be both retrospectively and prospectively useful throughout analysis.

Practically, data from a gallery walk can be used inductively and deductively in analysis. An inductive approach denoted the generation of new interpretative codes from the gallery walk data that were not already part of the coding schedule. A deductive approach related codes already in the coding schedule to the gallery walk data and looked to develop them based on participants’ discussions. For example, did they find this code in more or less RPs; did they connect it to specific icons that we have yet to interpret in this way; did they bring additional nuance and/or granularity to the code; did they make connections between codes, and so forth. Both new and enriched codes were applied to the RPs discussed by the participants and pulled back through the entire RP dataset as new lines of enquiry. The intention of this step in the gallery walk process was to ensure that the participants’ interpretations were rigorously integrated into the coding schedule and further analysis of the RPs, promoting its co-creation, expansion, and refinement.

## Discussion

**Fig 6:**
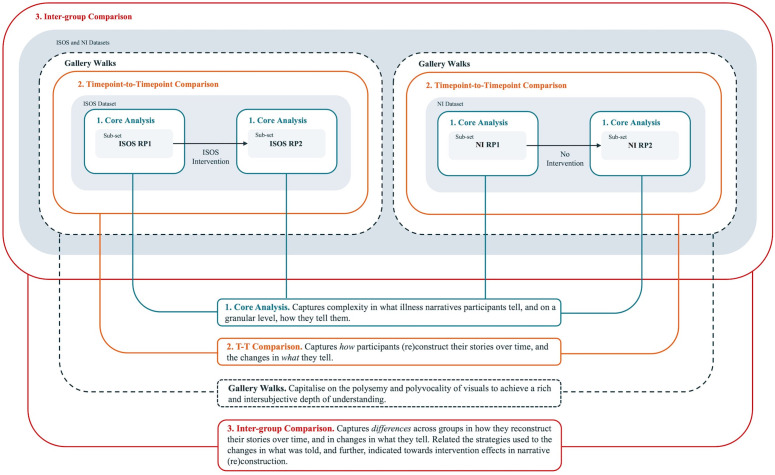
Summary of learnings from our multi-level systematic analysis of RPs.

This study departed from the proposition that visual representations of illness – and more specifically, RPs – may be powerful for exploring and narrating stories about life with incurable cancer. However, we understood that this potential may be limited by the continued precedence of verbal data in the analysis of visuals, alongside a lack of methodological guidance for analysing RPs themselves. Responding to this, we developed and presented a multi-level analysis process for RPs – without interviews – that moves from granular attention to icons, through to dataset-level comparisons over time, across groups, and across an intervention (Fig 6).

### Contributions of an analysis method for RPs to narrative research

Through developing our method, we achieved a layered analysis of visual narratives about life with cancer (Fig 6). This explored: (a) what – and, in granular terms, how – multidimensional stories are told by participants about their illness experiences through RPs (core analysis); (b) changes across the multiple dimensions of what participants are telling about their illness experiences, and strategies in how these stories are being (re)constructed, over time (T-T comparison); (c) differences across groups in the changes in what they tell about their illness experiences, and how they (re)construct stories over time; and (d) (narrative) intervention effects on participants’ illness narratives over time (inter-group comparison) [33].

Taken together, these analytic possibilities demonstrate a substantial scope in working directly with RPs themselves, and the kinds of narrative insights relevant to research and clinical practice that can be afforded. This reiterates findings that RPs can be useful for researching illness stories both at a single moment and longitudinally, while we add that such narratives can also be explored by analytically engaging with RPs themselves, as well as through comparisons across groups and interventions [4,14]. To our knowledge, this is the first approach that uses RPs to attempt these tasks. We do not suggest that RPs and their visual analysis supersede verbally oriented narrative approaches, nor claim that our findings are necessarily transferable given that the construction and interpretation of (visual) narratives are situated in and shaped by socio‑cultural context [3,39–41]. However, we do propose that analysing RPs as visual narratives in these ways augments existing methodological approaches and possible findings by bringing different narrative forms into analytical view. Our method begins to bridge the methodological gap of how to do so. We thus make – and see further value in – analysis‑focused contributions to methodological innovation in illness narrative research, responding to growing recognition that different methods afford and constrain different analytical insights and pathways. Future research could compare visual and verbal narrative (analysis) methods over time, across groups and interventions to explore what each approach affords and constrains, for whom, and in which contexts.

### Reframing visual analysis: A constructivist perspective

Concerns levied at visual analysis approaches, nonetheless, often centre on problems of misunderstanding, or mis- and over-interpretation [5,15,16,20–22]. From this perspective, a reliance upon verbal data provides a ‘neutralising’ or ‘gatekeeping’ strategy to verify or correct analysis in ways that stabilise validity – an approach similar to member checking [5,15,16,21,23,26]. Placing our method within a constructivist epistemological framework, we have alternatively worked to recognise that RPs are polysemic and meanings situated, co‑constructed and polyvocal. Rather than seeking a single definitive and standardised interpretation from the participant (or otherwise, e.g., through intercoder reliability measures), our approach values divergence, as well as the role of the researcher, as productive resources when rigorously working toward realising the potential contributions of RPs [11,26,36]. To support this, we have used a coding approach alongside gallery walks, second coders, and research team meetings, which invite multiple readings to enrich and deepen understanding. Elsewhere this has been described as processes of ‘crystallisation’ – enhancing rigour through the comprehensiveness of taking various angles of approach [26]. By making this move from verbal validation towards crystallisation, our work aligns with wider shifts within qualitative research to ‘shed the cobra effect’ – that is, to move away from well‑intentioned, but perhaps epistemologically mismatched, validation practices that may inadvertently narrow interpretive possibilities and contributions [26]. Future work might continue to reorient the analysis of RPs toward realising the potentials of constructivist, polysemic visual analysis.

### Researcher competencies in visual analysis

Constructively situating the role of the researcher in the analysis of RPs emphasises the importance of researcher competencies in visual analysis. Our method stands as an example of the expertise that researchers can bring when afforded to go beyond privileging the individual, verbal account, to achieve synthesis and abstraction from, and across, visual datasets [11,26,42]. However, within our method, we were habitually inspired by methods and software developed for verbal or text-based data. This reflects observations that even researchers who work overtly with visuals may feel more confident and familiar with verbal/text-based approaches in analytic practice [42]. Calls have thus been made to explore visual approaches to analysis itself, including co-creation of visuals in analysis phases [43]. At the same time, our research group had a relatively high level of ‘visual scientific literacy’ – combined visual-scientific skill – owing to the inclusion of members trained in the visual arts and experienced with RPs [44]. This may have been significant in our approach to RPs, shaping what was seen and which interpretive moves felt legitimate. This does not suggest that only researchers with arts-informed backgrounds can engage in visual analyses. Rather, we emphasise that the cultivation of tacit visual competencies should be considered in relation to the rigour, efficacy, and feasibility of our method, and as of interest to realising the potentials of RPs at large [44].

### Ethical and practical limitations

Beyond questions of validity, excluding participant interpretations of their own RPs from our method produces limitations. By taking this approach, participants’ own voices in the analysis were less directly foregrounded. This can sit in some tensions with ethical principles around hierarchy and co‑creation that underpin participatory arts‑based research practices [26]. Moreover, it excludes a ‘facet’ of perspectives that might further enrich and deepen understanding, with implications for rigour [26]. It also means that, despite RPs being considered an accessible method, our analysis approach did not account for potential challenges in the visual literacy of participants – a potentially important consideration for new contexts [45]. Whilst approaches, such as the gallery walks, worked to mitigate some of these concerns, future approaches might enhance our method through the ‘interpretative engagement framework’ – an approach which views both participant interpretation and systematic researcher interpretation as integral, partial, and complementary analytical stages [11]. Participant interpretations could be integrated into analyses in ways similar to those described for gallery walks [11]. Yet, any future inclusion requires intentional attention to, and further study of, how and why the relative weighting of visual and verbal data is handled [42]. Here researchers might pursue and investigate strategies in which neither is given more ‘value’, but rather systematically brought into productive dialogue.

Concerns about the feasibility of our visual analysis approach should also be acknowledged, and particularly its time‑consuming and laborious nature that limits uptake in large‑scale or clinical settings. How our approach differs in feasibility from more ‘standard’ interview or RP studies, and how efficiency relates to the rigour and contributions of qualitative research, remain open questions [46,47]. Whilst this is a site for future study through (comparative) feasibility work on RP use and analysis, it is also an opportunity for further analytical innovation [46,47]. Emerging work on AI‑supported qualitative analysis offers one avenue, provided these tools are used critically and epistemologically coherently, rather than assumed automatic solutions [48]. Despite this, forgoing (additional) requests for verbal elaboration may sometimes be appropriate and more feasible. This may be the case when, for example, such requests add inappropriately to participants’ time burden, when speech may be difficult or not possible (e.g., for some people living with head or neck cancers), or – as we argue – when experiences may still resist verbal narration.

Taking all this into account, we suggest future research should treat the inclusion of participant interpretations as a context‑dependent design choice, to be interrogated in relation to its value to the research, participant preferences and needs, and feasibility, rather than as a default requirement. Our method stands as an example of analysis approaches that make such choices feasible.

### Reporting a step-by-step multi-level analysis process for RPs

By reporting a technically detailed, step‑by‑step account of our multi-level analysis process, we aimed to show how visual analysis approaches might be conducted with sequential rigour and systematic care and to respond directly to the lack of transparent, practical guidance for undertaking such a task. A key contribution of our paper is its bid to fill this gap. Yet presenting – and visualising – our process in this stepwise and transparent way proved to be challenging, raising important limitations and questions about how analysis approaches might be reported. Particularly, we experienced friction in attempts to clarify and linearise processes that were also, in practice, iterative, intuitive, uncertain and shaped by various situated perspectives. This was not an easy task and produced a tension in our account: it risks presenting visual analysis as primarily ‘mechanical tasks’, rather than embodied, interpretative, and relational practice [49,50]. These are recognised challenges in reporting visual analyses – and ones that may be contributing to the lack of reporting on this topic [43]. Yet, they are also challenges faced by, and shaping the ‘anatomy’ of, qualitative methods sections more generally [50]. As such, we reiterate that our method is not, nor was, a formula or definitive protocol that guarantees success in new contexts [49]. Rather, it is a situated example that foregrounds specific choices and contingencies. This may be helpful and offer a transferable analytic scaffold, but it also requires adaptation, contestation, and development for different contexts. Researchers have therefore suggested such accounts may be considered ‘pedagogical launching pads’ [36,37]. Moreover, they ought to be recognised as interpretive acts that do not simply offer a neutral, transparent description of analysis, and which construct how visual analysis is understood, practiced, and rendered productive [50]. Future work should continue developing and sharing diverse practices for reporting visual analysis methods. These methodological accounts may serve as opportunities for explicitly reflexive contributions that can narrate complex journeys of interpretation and might further advance visual scientific discourse [11,50].

## Conclusion

This study introduced a multi-level, systematic process for analysing RPs themselves, as visual illness narratives. In doing so, we offer, to our knowledge, the first step-by-step visual analysis method for investigating how people (re)create stories about life with an incurable illness at one moment in time, longitudinally, across groups and interventions. The analytic possibilities of our method demonstrate the substantial scope of working directly with RPs, complementing verbally oriented narrative (analysis) approaches by bringing different narrative forms and possible insights into analytical view. By analytically foregrounding RPs themselves, we encourage researchers to assess assumptions of verbal validation and realise the potentials of a constructivist, polysemic understanding of visual narratives that capitalises on the situated co-construction of meanings, as well as researcher competencies. Rather than being understood as a methodological requirement, we also suggest that the inclusion and weighting of verbal participant data be considered as a context‑dependent design choice in RP research, contingent upon epistemic aims and frameworks, participant preferences, competencies and needs, as well as ethical principles and feasibility. The presentation of our stepwise method is offered as scaffolding example for researchers, inviting adaptation and development in new contexts. Continuous reflexive attention to analytic processes in visual methods at large may open innovative directions in illness narrative inquiry, and beyond.
